# Robotic Quad Zygomatic Implant Surgery Accuracy Ex Vivo With a New Semi‐Autonomous Optically‐Driven Haptic System

**DOI:** 10.1111/cid.70155

**Published:** 2026-04-28

**Authors:** Alessandro Pozzi, James Chow, Andrea Laureti, Lorenzo Arcuri, Ilaria Cammarota, Alessandra Nardi, Hom‐Lay Wang

**Affiliations:** ^1^ Department of Clinical Science and Translational Medicine University of Rome Tor Vergata Rome Italy; ^2^ Department of Restorative Sciences Augusta University Augusta Georgia USA; ^3^ Department of Restorative Dentistry and Biomaterials Sciences Harvard School of Dental Medicine Boston Massachusetts USA; ^4^ Brånemark Osseointegration Centre Hong Kong People's Republic of China; ^5^ Department of Chemical Science and Technologies University of Rome Tor Vergata Rome Italy; ^6^ Division of Fixed Prosthodontics and Biomaterials University Clinics for Dental Medicine, University of Geneva Geneva Switzerland; ^7^ Department of Periodontics and Oral Medicine School of Dentistry, University of Michigan Ann Arbor USA; ^8^ Department of Life Science, Health and Health Professions Link Campus University Rome Italy; ^9^ Department of Mathematics University of Rome “Tor Vergata” Rome Italy

## Abstract

**Objectives:**

To evaluate ex vivo accuracy of robotic computer‐assisted implant surgery (r‐CAIS) for quad zygomatic implant (ZI) placement with a novel semi‐autonomous, optically‐driven, haptic‐assisted system.

**Methods:**

Two expert operators placed ZIs in human cadavers. Accuracy was evaluated by superimposing pre‐ and post‐operative cone‐beam computed tomography (CBCT) scans, measuring linear (mm) on the *x*‐, *y*‐, and *z*‐ axes and angular (degrees) deviations. Sample size was calculated. The main analysis focused on composite outcomes (global platform, global apical, and angular deviation), while extended analyses examined deviations on individual spatial axes (buccal–lingual, mesial–distal, depth, and non‐depth) for both implant platform and apex. Three multivariate models were executed on global platform, global apical, and angular deviation considering implant position (anterior vs. posterior) and quadrant (left vs. right).

**Results:**

Forty ZIs were placed in 10 human cadavers. The mean global deviations at implant platform and apex were 1.16 mm (standard deviation (SD) 0.47) and 1.51 mm (SD 0.70), with mean angular deviation of 1.04° (SD 0.65°). In the multivariate models, implant position was found to be significant for global platform deviation (*p* = 0.0218), with posterior implants performing better, and for angular deviation (*p* = 0.0465), with anterior implants performing better.

**Conclusion:**

The optically driven haptic‐assisted robotic system demonstrated high accuracy in quad ZI surgery, with all implants placed without intraoperative complications or significant errors impacting adjacent anatomical structures. Mean global linear deviations of 1 and 1.5 mm at platform and apex, and 1° of angular deviation were experienced. Further research is necessary to validate these promising results in vivo.

## 1 Introduction

Zygomatic implants (ZIs) offer a reliable solution for the rehabilitation of severely atrophic maxillae, eliminating the need for bone grafting procedures [1, 2, 3]. They provide stable anchorage for fixed prosthetic rehabilitation through a less invasive surgical approach compared to conventional bone augmentation techniques [4, 5, 6, 7]. A systematic review and meta‐analyses by Brennand‐Roper assessed the predictability of ZI therapy, reporting survival rates exceeding 95% over extended follow‐up periods [8].

In cases of extreme maxillary atrophy, where both anterior and posterior bone volumes are insufficient for conventional implant placement, the quad zygoma protocol—based on the bilateral placement of four zygomatic implants—has been proposed as a graftless solution for full‐arch rehabilitation [9]. This approach relies on cross‐arch stabilization and allows immediate or early loading in highly compromised anatomical conditions.

Long‐term clinical evidence has demonstrated high implant survival rates for the quad zygoma approach, with pooled survival values approaching 96%–98% over extended follow‐up periods [10, 11]. However, systematic reviews and consensus reports have consistently emphasized that such predictability is achieved at the expense of increased surgical complexity and a non‐negligible risk of biological and technical complications, particularly when implant positioning accuracy is compromised [11, 12].

Owing to the extreme implant length, complex three‐dimensional trajectories, and close proximity to critical anatomical structures such as the orbit and maxillary sinus, quad zygomatic implant surgery represents one of the most demanding scenarios in implant dentistry, in which even minimal deviations may result in clinically relevant apical displacement. In this clinical context, achieving a high level of three‐dimensional accuracy becomes a critical requirement, as even minimal deviations may have relevant clinical consequences [11, 13, 14, 15, 16, 17].

Digital technologies can enhance the accuracy of ZI placement through techniques like static (s‐CAIS), dynamic (d‐CAIS), and robotic (r‐CAIS) computer‐aided implant surgeries [18, 19, 20, 21, 22, 23]. Static‐CAIS employs advanced computer‐aided design/computer‐aided manufacturing (CAD/CAM) surgical templates [24]. These templates are designed to guide the drilling according to the precise implant position planned through Cone‐Beam Computed Tomography (CBCT) images, standard tessellation language (STL) data, and dedicated implant planning software. The placement of ZIs using s‐CAIS requires long sleeves on the surgical guide to account for the potential deviations from the long ZI drills; these can make templates bulky, potentially impacting the intraoral working space [22]. To address these limitations, dynamic CAIS or navigation implant surgery was developed.

D‐CAIS uses camera‐based live patient and contra‐angle tracking, enabling real‐time visualization of the drilling and implant placement trajectory, guided by CBCT imaging data [23]. It allows continuous visual inspection of the implant site, live tracking, full guidance, and “real‐time” adjustments if deviations occur, without a template obstructing the surgical field or hampering soft tissue management. Real‐time visualization provided by d‐CAIS can improve intraoperative safety in the confined zygomatic surgical field [25].

While both s‐CAIS and d‐CAIS significantly reduce deviations compared to free‐hand surgery, [26, 27] each has specific limitations that can affect clinical accuracy [28, 29, 30] Robotic systems have been progressively introduced into implant surgery to overcome these limitations, potentially eliminating human‐related surgical deviations. R‐CAIS incorporates advanced technologies, including artificial intelligence (AI), augmented reality, machine vision, multi‐sensor data fusion, and three‐dimensional (3D) graphical visualization [31, 32].

Yang et al. [33] classified robotic systems by their level of autonomy, ranging from Level 0 to Level 5. Level 0 includes systems with no autonomy, such as tele‐operated robots that follow the user's commands; even systems with motion scaling fall into this category, as they replicate the surgeon's movements without independent decision‐making. Level 1 involves robotic assistance, offering mechanical guidance while the operator maintains continuous control. Level 2 refers to task autonomy, where the robot performs specific tasks autonomously after initiation by a human; here, the surgeon exercises discrete rather than continuous control. Higher levels—3 (conditional autonomy), 4 (high autonomy), and 5 (full automation)—are future goals, requiring advanced AI for real‐time decision‐making, adaptability, and independent execution of complex surgical procedures [33, 34, 35].

The primary aim of this ex vivo study was to evaluate the accuracy of ZI positioning using a novel semi‐autonomous dental implant robotic system. Secondary aims include assessing how implant position (anterior vs. posterior) and quadrant (left vs. right) affect accuracy. The null hypothesis was that there would be no significant differences in overall linear and angular deviations among these variables.

## 2 Materials and Methods

The ex vivo study was strictly conducted following the legal, ethical, and procedural recommendations recently stated by the American Association for Anatomy [36].

**FIGURE 1:**
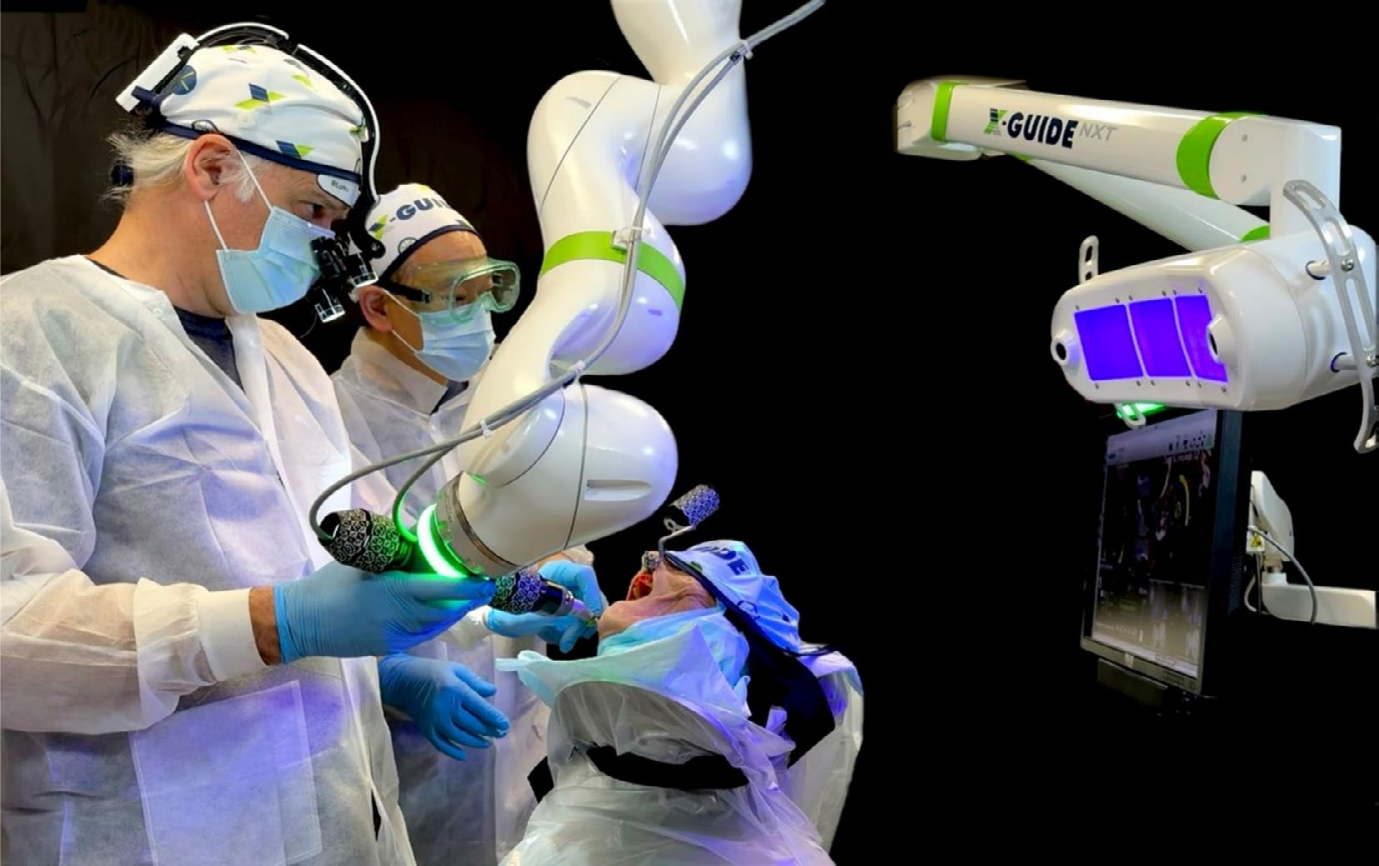
Intra‐operative picture of the novel semi‐autonomous, optically‐driven, haptic‐assisted robotic system. In the picture is visible the 7‐joint light weight robotic arm and, on the right, the dynamic navigation's cameras. The system's optical tracking detects and triangulates the patterned part attached to the specimen and to the handpiece.

Ten fresh‐frozen human cadaver heads were selected for treatment with four ZIs (NobelZygoma 0°CC TiUltra, Nobel Biocare) using a novel level 1 semi‐autonomous optically driven haptic‐assisted robot (XguideRobotics, X‐Nav Technologies). (Figure 1) Each specimen underwent a high‐speed CBCT scan (i‐CAT FLX V17 Dexis) with a large field of view (FOV 160 mm height, 130 mm width) and a voxel size of 0.25 mm. A detailed 3D surgical plan of the quad‐zygoma surgery was generated using dedicated software (DTX Studio, DEXIS), and the implant plan was exported into the robotic system (X‐Guide, X‐Nav Technologies) to execute the r‐CAIS.

### 2.1 Robotic System Description

The novel Level 1 semi‐autonomous, optically‐driven haptic‐assisted robotic system is comprised of two main components (1) a robotic arm with a control system and (2) a dynamic navigation system. This robotic arm features a 7‐joint lightweight arm with integrated joint torque and joint position sensing, enhancing responsiveness to hand‐guided inputs and obstacle sensitivity. The navigation system combines an optical tracking system with a suite of tool trackers, calibration and patient registration algorithms, implant planning tools and on‐screen visualizations of the live surgical environment. The system's optical tracking uses a navigation assembly equipped with lights and multiple cameras to detect and triangulate a patterned part attached to the patient to reference and compensate for the position, orientation, and movement of the targeted patient anatomy. A similar patterned part is attached to the dental handpiece to track its position and orientation relative to the patient tracker. During r‐CAIS, the surgical handpiece and its drill tracker are mounted at the end of the robotic arm. The control system combines positional and orientational error from both the optical tracking system and the robot arm's sensors, enhancing visual guidance. This optical tracking data is provided visually to the surgeon and is integrated into the robotic arm system, delivering haptic feedback. This guides the drill to the 3D planned position and adds stability against variations in bone density, patient movement, or other disturbances encountered during drilling.

The robotic system provides 3 distinct haptic feedback modes: (1) Free mode, allowing minimal resistance, enabling the surgeon to maneuver the surgical instrument similarly to freehand surgery, (2) Haptic assistance mode activates by pressing a button on the robot, gradually aligning the drill with the desired implant axis while remaining compliant to the surgeon's inputs or to encountered obstructions during motion, and (3) Haptic resistance mode, which allows generally free motion when moving the surgical instrument toward the desired implant axis but firmly resists deviations away from this path. Buttons on the robot allow switching between these modes, e.g., after each drill in the sequence reaches its final depth, or to enter haptic mode, e.g., after a line‐of‐sight disruption, or after manually switching to free mode.

### 2.2 Surgical Planning

**FIGURE 2:**
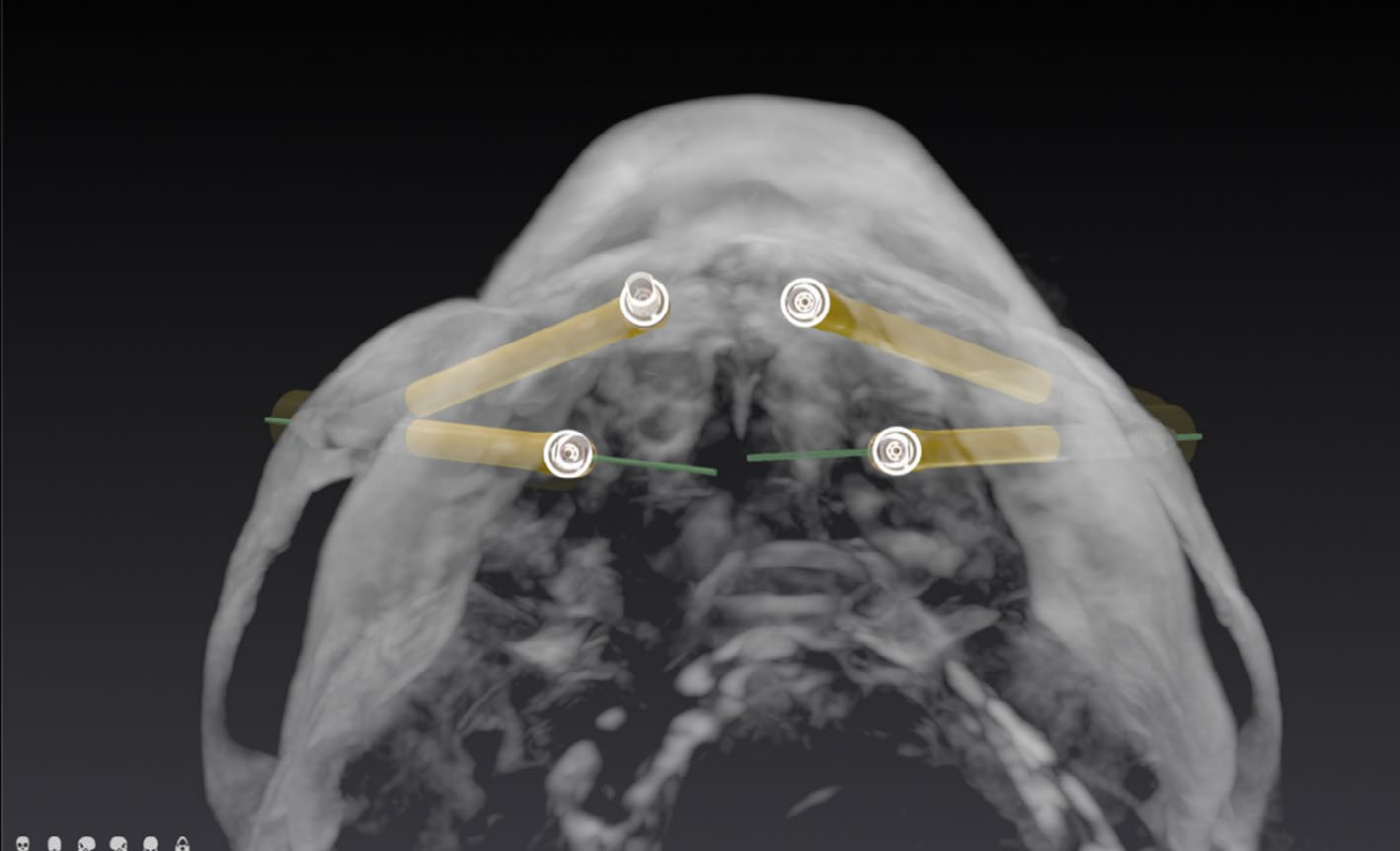
Virtual surgical planning showing prosthetic‐driven quad zygoma configuration aimed at maximizing anterior–posterior (AP) spread through combined anterior and posterior zygomatic implant placement.

**FIGURE 3:**
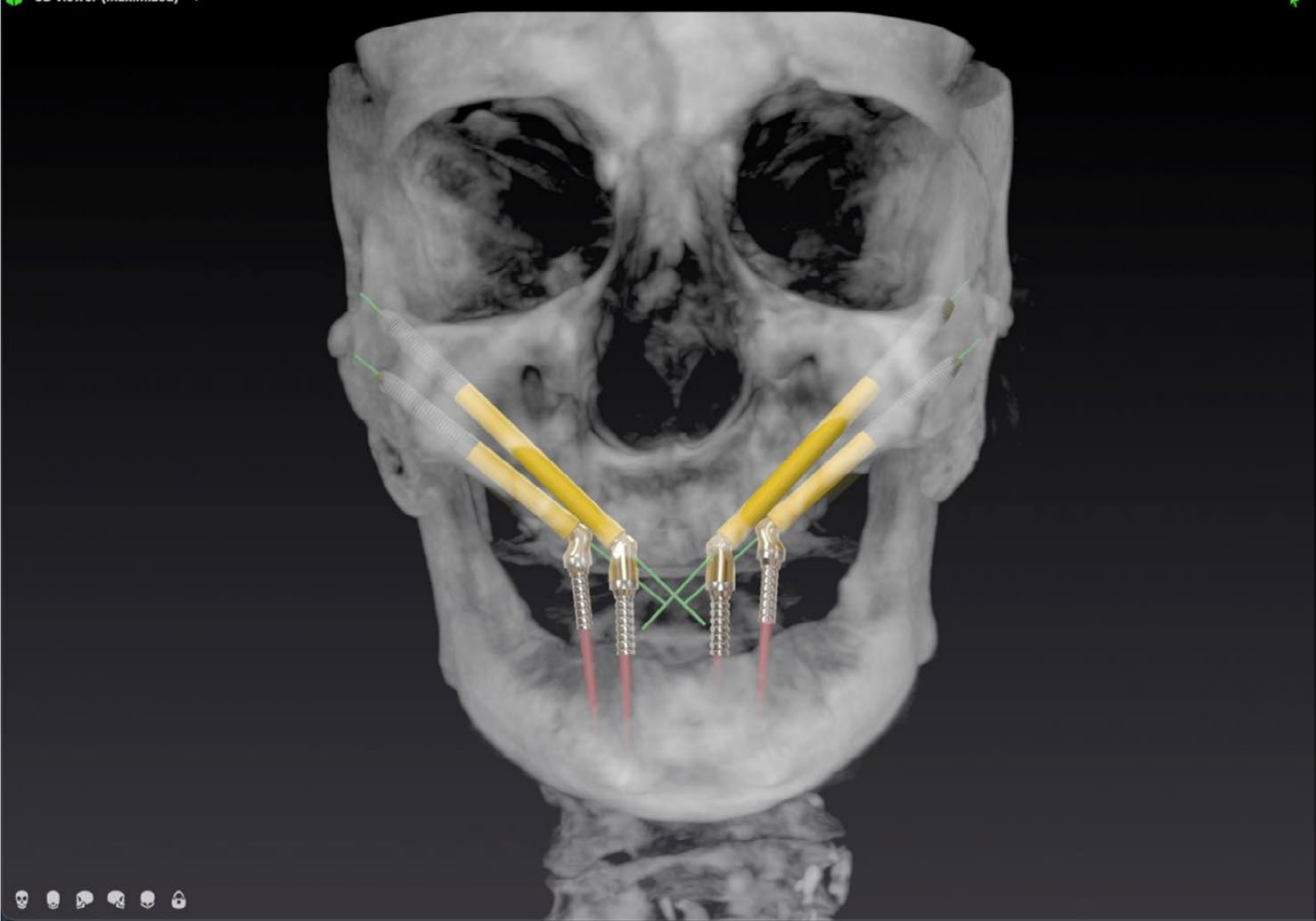
3D virtual surgical planning of quad zygoma implants based on CBCT data, defining implant trajectories according to anatomical constraints and optimal zygomatic apical anchorage.

Virtual surgical planning was performed using an implant planning software (DTX Studio, Dexis) to generate a 3D quad zygoma plan based on CBCT data. In accordance with established zygomatic implant concepts, planning followed prosthetic‐driven principles at the arch level, aiming to achieve a wide anterior–posterior (AP) spread through the combined placement of anterior and posterior zygomatic implants. (Figure 2) The definition of individual implant trajectories was subsequently guided by anatomical constraints. Anterior zygomatic implants were planned with coronal entry points in the anterior maxillary–palatal region and oblique trajectories directed toward the anterior portion of the zygomatic body, whereas posterior zygomatic implants were planned with entry points in the posterior maxilla and more vertical trajectories engaging the posterior zygomatic buttress. In all cases, implant paths were optimized to achieve remote apical anchorage within the zygomatic bone, which is considered essential for primary stability and successful immediate loading in quad zygoma rehabilitations. According to Hung et al. [37] on optimal positioning of zygomatic implants, the most favorable apical anchorage for quad cases is in specific regions of the zygomatic bone (A3 and B1), where maximal bone‐to‐implant contact can be obtained while minimizing the risk of penetration into critical anatomical structures, such as the orbital cavity and infratemporal fossa. (Figure 3).

### 2.3 Registration Protocol

An AI‐driven algorithm was employed to assist in placing 3 self‐drilling titanium bone screws (1.5 mm diameter, 4–5 mm in length) (Maxdrive screws, KLS Martin SE & Co.) into the maxillary bone before the CBCT scan, serving as fiducial landmarks for automatic registration. A fiducial‐based registration protocol, driven by an AI algorithm (X‐mark, X‐Nav Technologies), automatically identifies these screws within the DICOM anatomy, pairing with virtual geometries. A dedicated registration probe then aligns the virtual geometries with the real anatomy by touching each screw head in the mouth, synchronizing the CBCT and planned implants with the specimen's anatomy.

The three fiducial screws were precisely positioned during the planning phase to achieve a wide equilateral geometry triangular configuration. This arrangement was obtained by placing one fiducial in proximity to the nasal spine region and the remaining two posteriorly in the maxillary tuberosity area, as far posterior as possible and on the palatal side. This location was selected to take advantage of the thicker cortical plate, ensuring adequate primary stability while maintaining a safe distance from the zygomatic implant insertion site at the alveolar crest. Such a distribution provided an optimal spatial arrangement by maximizing the anterior–posterior spread among the fiducials, which represents a fundamental prerequisite for accurate registration and for minimizing software‐dependent deviations during drilling across the extensive maxillary–zygomatic region [38].

All fiducial screws demonstrated adequate mechanical stability within the bone, as confirmed after tapping at the time of placement prior to CBCT acquisition and subsequently verified intraoperatively before the registration procedure.

### 2.4 Surgical Protocol

Calibration of the surgical handpiece and patient tracking arrays was performed prior to surgery. The handpiece calibration determined the relationship between the geometry of the handpiece tracking array and the axis of the drill. The patient tracking array was connected to the bone with a dedicated metal arm secured with self‐tapping bone fixation screws. The surgical handpiece and patient tracking arrays must be within the line of sight of the overhead stereo cameras to be accurately tracked on the monitor. Hence, a link between the preoperative planning and the tracking coordinate system is automatically generated. The stereo tracking algorithm continuously triangulates the arrays precise position and orientation in a common coordinate frame during the surgery. The drill body and tip's dynamic connection to the patient's CBCT anatomy and pre‐planned implant coordinates are displayed with high magnification on a dedicated screen. All ZIs were placed with the robotic surgery system (X‐Guide Robotics, X‐Nav Technologies) following the manufacturer's drilling protocol. Two experienced clinicians (J.C., A.P.) performed all the surgical procedures after 2 days of robotic navigation training and completing 20 robotic surgeries each. The system consistently tracked motion between the skull‐anchored and the robotic handpiece reference frames, aligning surgical drilling with 3D implant coordinates through real‐time guidance.

### 2.5 Accuracy Evaluation

**FIGURE 4:**
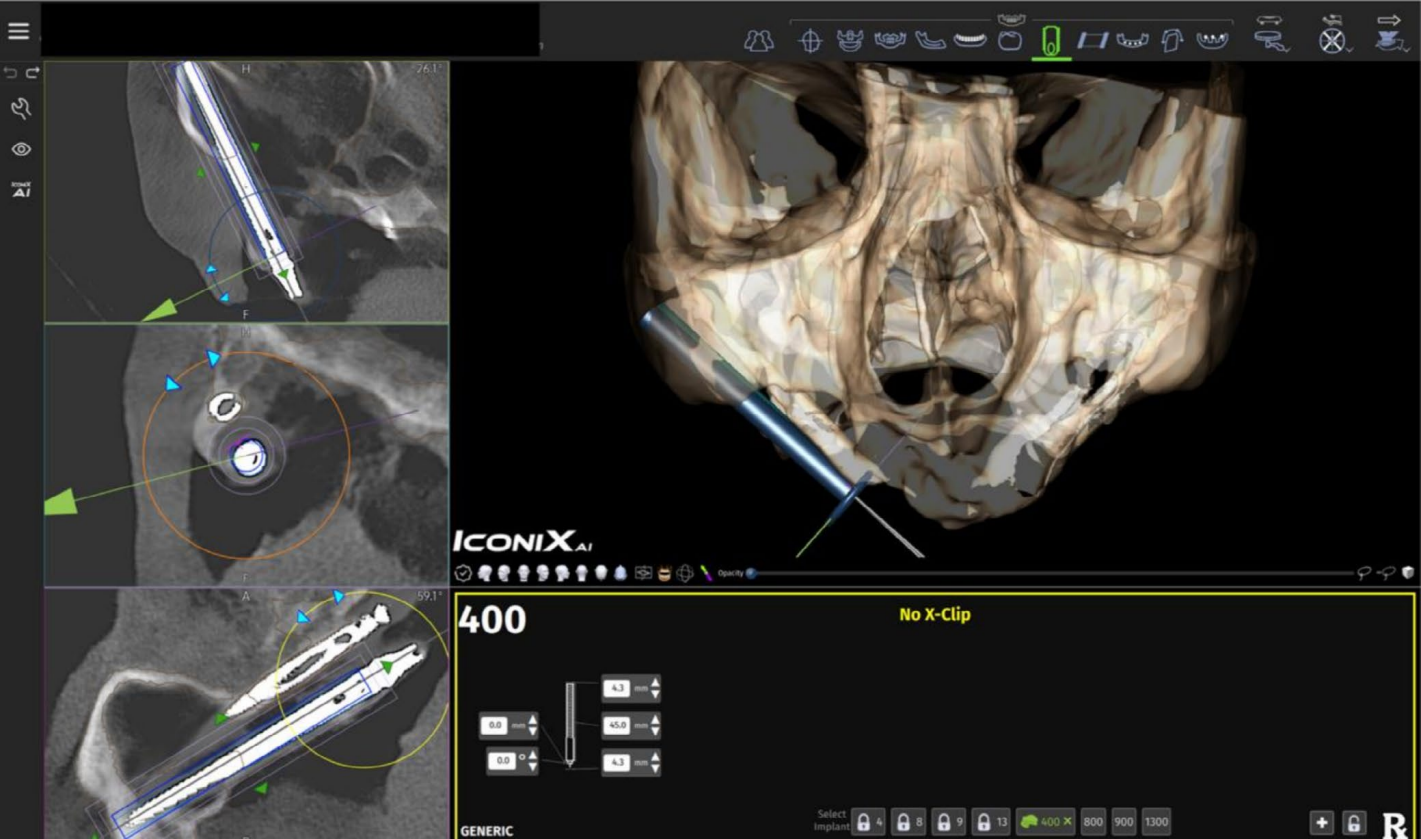
Accuracy evaluation: Implant library geometries were aligned in the postoperative cone beam computed tomography (CBCT) to the respective images of the placed implants.

Post‐surgery, a CBCT scan matched the pre‐operative scan's FOV and resolution. To evaluate accuracy, two mesh files (STL format) were superimposed: one representing the implant plan, and the other generated from the post‐operative CBCT scan. The first mesh, including xyz implant coordinates, was exported as an STL file from the X‐Guide software (X‐Guide, X‐Nav Technologies). The implant characteristics (diameter, length, and position) were extracted using the X‐Guide Accuracy Analysis tool. The second mesh file was generated by uploading the post‐operative CBCT scan into the X‐Guide software. (Figure 4) The digital representations of the placed implants were aligned with their corresponding radiopaque images and exported as an STL file. Using the “point‐based gluing” function in MeshLab (Visual Computing Lab, ISTI‐CNR), preoperative and postoperative meshes were merged by selecting anatomical landmarks, ensuring precise alignment. Anatomical landmarks, such as thick cortical bone, were selected on both meshes to ensure precise alignment. Visual feedback was used to refine the superimposition, achieving a “marble‐like” appearance, and the aligned file was exported in MLP format (MeshLab file format) and opened in X‐Guide. The “X‐Guide Accuracy Analysis” tool analyzes the three files (preoperative plan, postoperative plan, and merged file), generating a .txt file with implant deviation data. This data is processed in GNU Octave (GNU General Public License, University of Texas) and converted into an Excel spreadsheet (Microsoft), detailing angular (°) and linear (mm) deviations for each implant.

### 2.6 Outcomes

**FIGURE 5:**
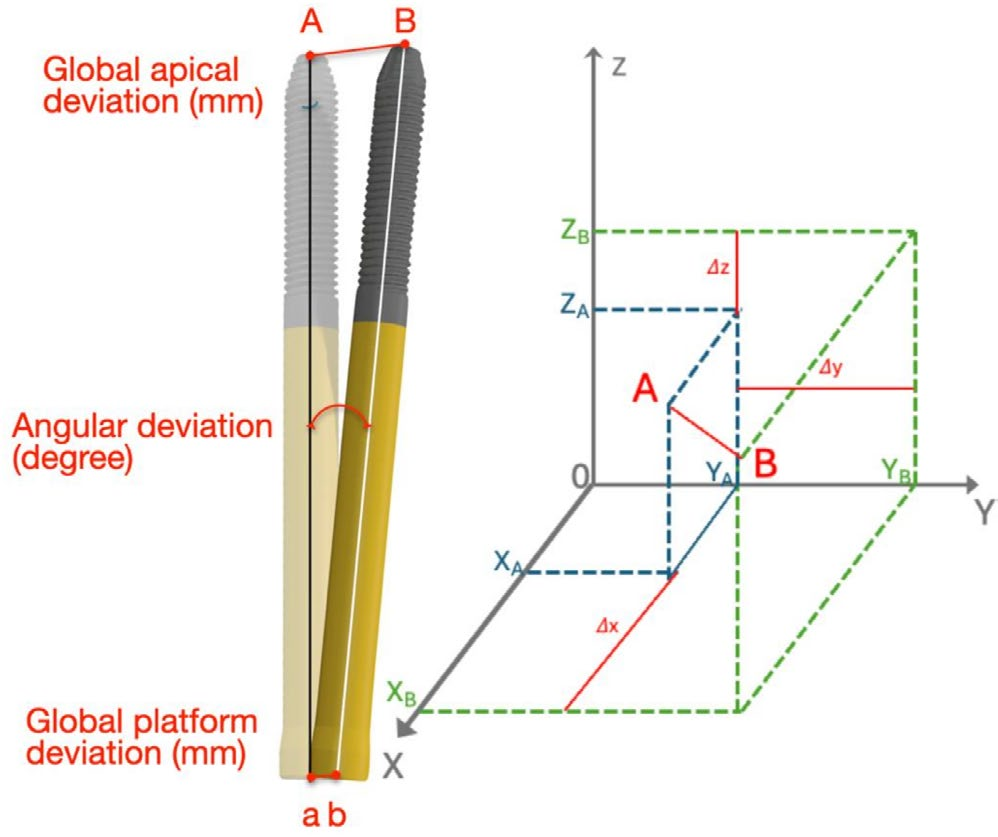
3D and angular deviation assessment. The global linear deviation was calculated as the distance between the centers of the platform (a, b) and apex (A, B) of the planned and placed implants. That distance was projected onto the three spatial axes to calculate linear deviations (ΔX, ΔY, ΔZ). The angular deviation was calculated as the angle formed by the axes of the implants.

Primary outcome variables were global platform, global apical, and angular deviation. Additionally, 3D deviations at the implant platform and apex were analyzed in the buccal‐lingual (*x*‐axis), mesial‐distal (*y*‐axis), and vertical (*z*‐axis) directions. Definitions and graphical visualization of the investigated outcomes are provided in Table 1 and Figure 5, respectively.

**TABLE 1:** Investigated outcomes.

Outcome	Definition
Global Platform deviation (mm)	Overall 3‐dimensional distance between the platform centroids of the planned and placed implants.
Platform bucco‐lingual (BL) deviation (mm)	Buccolingual distance between the platform centroids of the planned and placed implants on the *x*‐axis
Platform mesio‐distal (MD) deviation (mm)	Mesiodistal distance between the platform centroids of the planned and placed implants on the *y*‐axis
Platform Depth deviation (mm)	Depth distance between the platform centroids of the planned and placed implants on the *z*‐axis
Platform non‐depth deviation (mm)	Distance between the platform centroids of the planned and placed implants on the *x*‐ and *y*‐axis.
Global apical deviation (mm)	Overall 3‐dimensional distance between the apex centroids of the planned and placed implants
Apical BL deviation (mm)	Buccolingual distance between the apex centroids of the planned and placed implants on the *x*‐axis
Apical MD deviation (mm)	Mesiodistal distance between the apex centroids of the planned and placed implants on the *y*‐axis.
Apical Depth deviation (mm)	Depth distance between the apex centroids of the planned and placed implants on the *z*‐axis.
Apical non‐depth deviation (mm)	Distance between the apex centroids of the planned and placed implants on the *x*‐ and *y*‐axis.
Angular deviation (°)	Angle formed by the vertical axes of the planned and placed implants

### 2.7 Statistical Analysis

The sample size of 40 implants guaranteed a 95% confidence interval with a width of 0.38 mm for global platform deviation assuming a standard deviation of 0.50, of width of 0.52 mm for global apical deviation assuming a standard deviation of 0.67, of width of 0.46 mm for angular deviation assuming a standard deviation of 0.61. The probability that confidence intervals will not exceed the declared widths is 0.95.

Assumed standard deviations were based on Deng et al. [17] Note that the overall probability of achieving the desired precision and capturing the true mean for each confidence interval is 0.90.

Continuous variables were summarized by mean, standard deviation, minimum and maximum value. Kernel density estimates were used to describe empirical distributions. Confidence bands are based on 1000 bootstrap replicates.

Multivariable analysis was based on the mixed linear model. Three different models were fitted assuming global platform, global apical, and angular deviation as response variables. In each model, implant position (anterior vs. posterior) and quadrant (left vs. right) were modeled as fixed effects.

The random effect incorporates the correlations of all the measurements taken on the same cadavers, and it was modeled by introducing a random intercept. No a priori structure was assumed for the covariance matrix.

All analyses were undertaken using SAS software version 9.4 (SAS Institute, Cary, NC, USA) and R version 4.4.

## 3 Results

**FIGURE 6:**
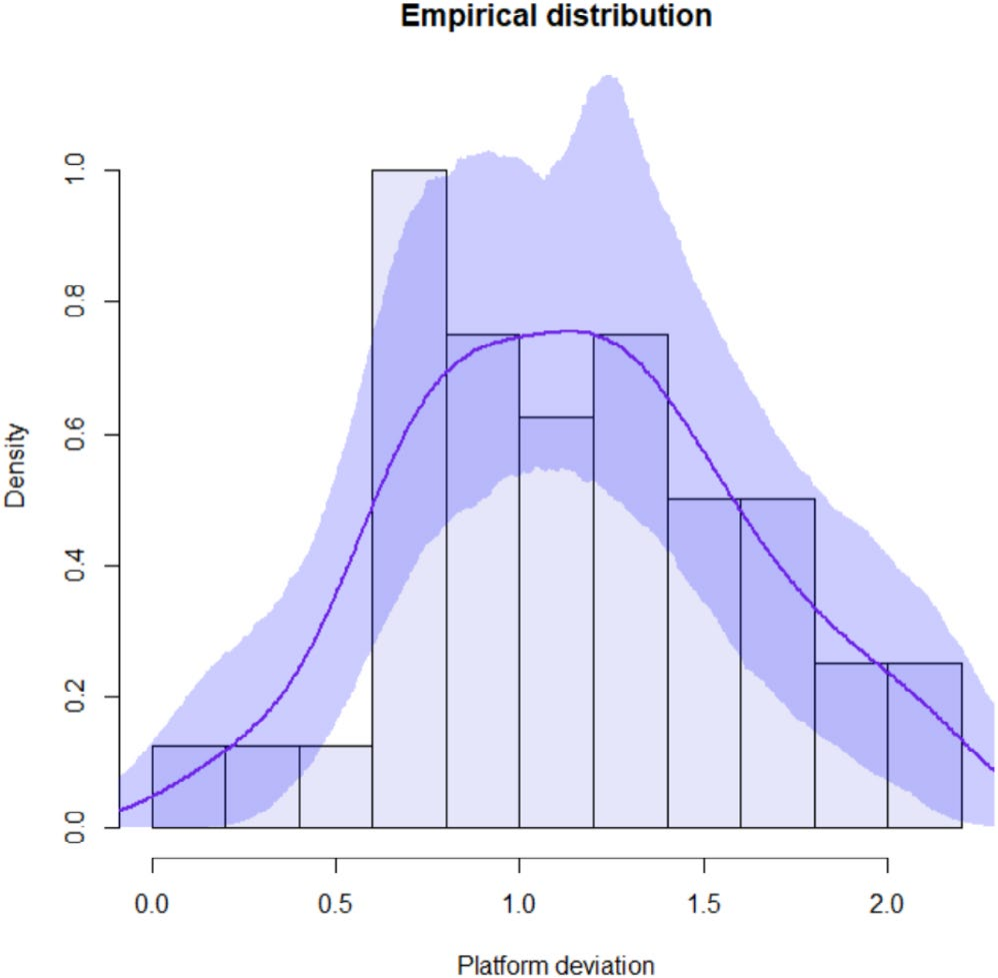
Platform deviation distribution curve with confidence intervals.

**FIGURE 7:**
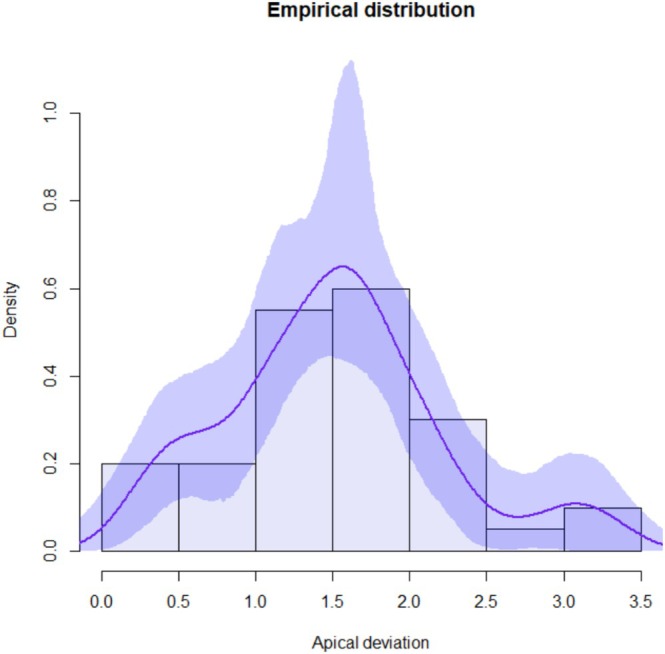
Apical deviation distribution curve with confidence intervals.

**FIGURE 8:**
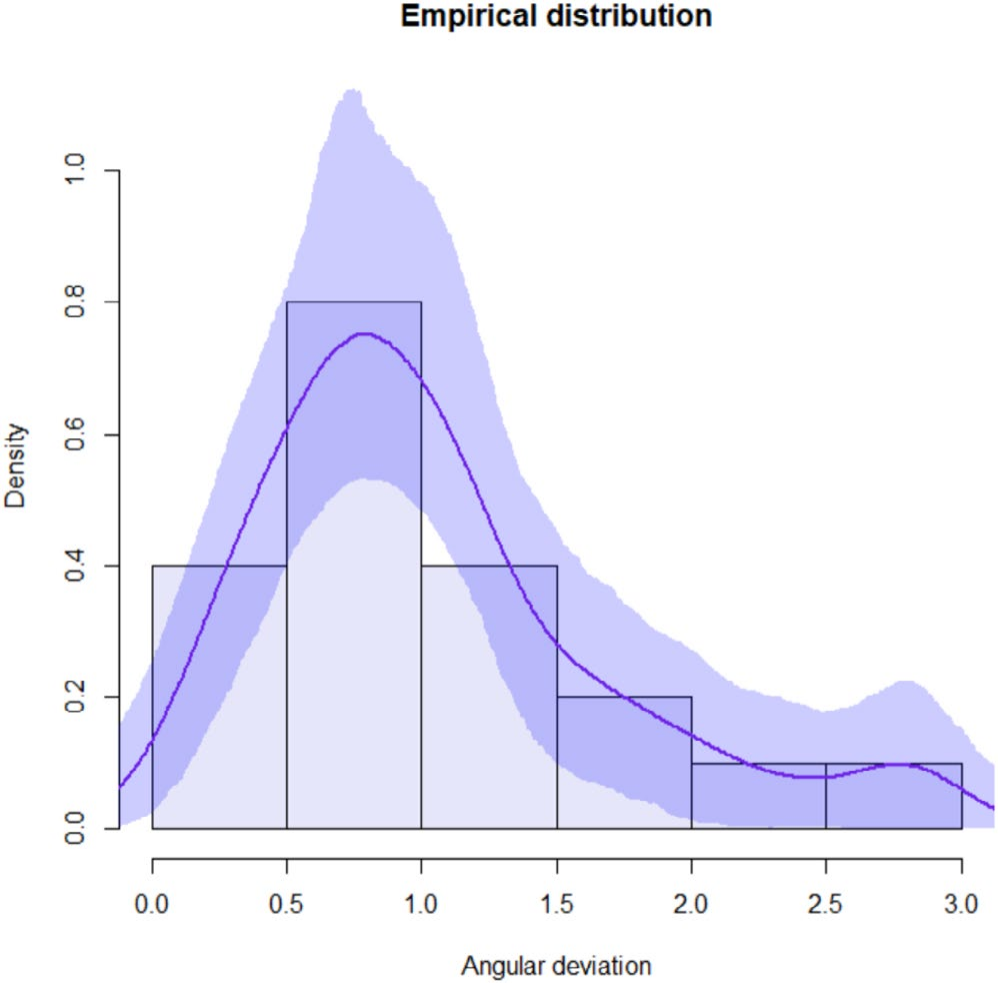
Angular deviation distribution curve with confidence intervals.

Forty ZIs were placed in 10 cadaver heads. A total of 10 complete arches were treated with quad zygoma surgical technique. The mean global platform and apical deviations were 1.16 mm (standard deviation (SD) 0.47) and 1.51 mm (SD 0.70), respectively. The mean angular deviation was 1.04° (SD 0.65). The maximum global deviation recorded was 3.24 mm at the apex. Complete descriptive analysis is reported in Table 2. Empirical distributions with confidence intervals related to platform, apical, and angular deviations are shown in Figures 6, 7, 8.

**TABLE 2:** Descriptive analysis.

	Global platform deviation (mm)	Global apical deviation (mm)	Angular deviation (degree)	Platform BL (X) deviation (mm)	Platform MD (Y) deviation (mm)	Platform Depth (Z) deviation (mm)	Apical BL (X) deviation (mm)	Apical MD (Y) deviation (mm)	Apical depth (Z) deviation (mm)
Mean	1.16	1.51	1.04	−0.36	−0.46	−0.25	−0.13	−0.53	−0.27
SD	0.47	0.70	0.65	0.61	0.68	0.71	1.01	0.84	0.71
Min	0.18	0.32	0.06	−1.55	−1.73	−1.46	−2.19	−2.69	−1.47
Max	2.13	3.24	2.86	1.24	1.56	1.94	2.2	1.04	1.94

**FIGURE 9:**
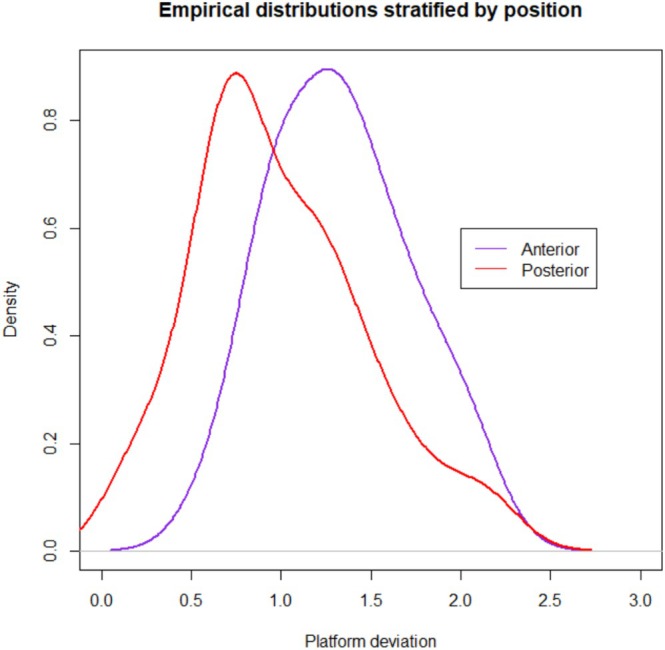
Empirical distributions of global platform deviations stratified per implant position (anterior vs. posterior).

**FIGURE 10:**
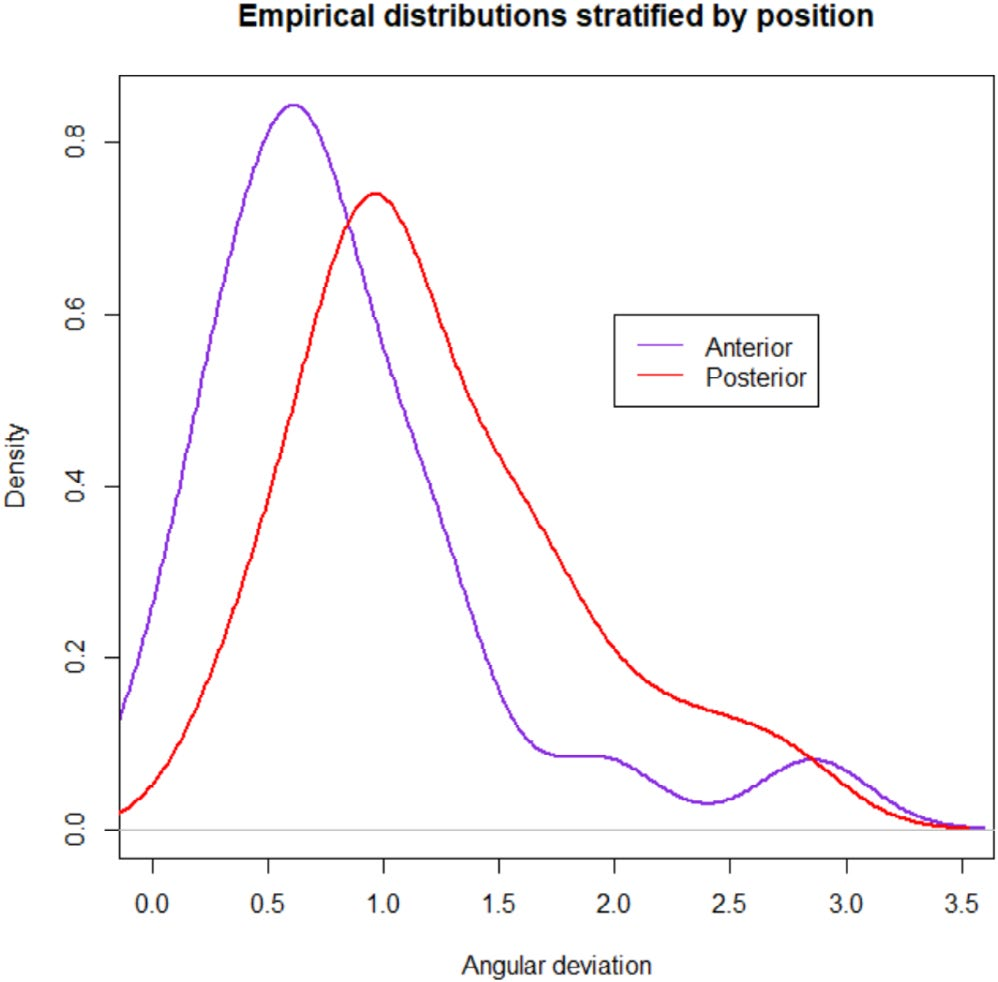
Empirical distributions of angular deviations stratified per implant position (anterior vs. posterior).

Three multivariable models were fitted for global platform, global apical, and angular deviation considering the fixed effect of implant position (anterior vs. posterior) and quadrant (left vs. right). Tables 3, 4, 5, 6, 7, 8 report estimates of fixed effects and estimated expected deviations. Implant position was found to be significant for global platform deviation (*p* = 0.0218), with posterior implants performing better. Related empirical distributions of global platform and angular deviations stratified per implant position (anterior vs. posterior) are shown in Figures 9 and 10. Implant position was found to be significant also for angular deviation (*p* = 0.0465), with anterior implants performing better. None of the analyzed variables resulted significant for global apical deviation. Posterior implants were divided into two groups according to the sinus trajectory. Eleven implants had an extrasinus (ES) and 9 an intrasinus (IS) trajectory. The mean ES versus IS global platform and apical deviations were 0.85 mm (SD 0.36) versus 1.26 mm (SD 0.49), and 1.29 mm (SD 0.52) versus 1.54 mm (SD 0.83), respectively. The mean ES versus IS angular deviation was 1.39° (SD 0.48) versus 1.06° (SD 0.74). Sinus trajectory was not considered in the multivariate models as it is evaluated only on posterior implants.

**TABLE 3:** Estimates of fixed effects on global platform deviation from the linear mixed model.

Effect	Estimate	Standard error	*t*	*p*
Intercept	1.05	0.12		
Implant position (Anterior)	0.34	0.14	2.43	0.0218
Quadrant (Left)	−0.13	0.14	−0.90	0.3752

*Note:* Reference levels for implant position and quadrant were Posterior and Right, respectively.

**TABLE 4:** Estimated expected global platform deviation (mm) by implant position and quadrant.

Expected platform deviation	Right	Left
Anterior	1.30 (1.15, 1.64)	1.27 (1.02, 1.51)
Posterior	1.05 (0.81, 1.30)	0.93 (0.68, 1.18)

**TABLE 5:** Estimates of fixed effects on global apical deviation from the linear mixed model.

Effect	Estimate	Standard error	*t*	*p*
Intercept	1.53	0.20		
Implant position (Anterior)	0.21	0.21	1.01	0.3212
Quadrant (Left)	−0.26	0.21	−1.23	0.2277

*Note:* Reference levels for implant position and quadrant were Posterior and Right, respectively.

**TABLE 6:** Estimated expected global apical deviation (mm) by implant position and quadrant.

Expected apical deviation	Right	Left
Anterior	1.74 (1.34, 2.14)	1.49 (1.08, 1.89)
Posterior	1.53 (1.13, 1.93)	1.27 (0.87, 1.68)

**TABLE 7:** Estimates of fixed effects on angular deviation from the linear mixed model.

Effect	Estimate	Standard error	*t*	*p*
Intercept	1.31	0.18		
Implant position (Anterior)	−0.40	0.19	−2.08	0.0465
Quadrant (Left)	−0.14	0.19	−0.72	0.4797

*Note:* Reference levels for implant position and quadrant were Posterior and Right, respectively.

**TABLE 8:** Estimated expected angular deviation (degrees) by implant position and quadrant.

Expected angular deviation	Right	Left
Anterior	0.90 (0.55, 1.27)	0.77 (0.41, 1.13)
Posterior	1.31 (0.95, 1.67)	1.17 (0.81, 1.53)

## 4 Discussion

Zygomatic implants (ZIs) pose unique challenges due to their length and proximity to critical anatomical structures such as the orbit and the maxillary sinus [13, 14]. Even small deviations can cause significant displacement at the apex [20]. Thus, the accuracy of the robotic system is crucial in this context. The primary aim of this ex vivo study was to assess the accuracy of quad ZI surgery using a novel Level 1 semi‐autonomous, optically‐driven, haptic‐assisted dental implant robotic system. Within the limitations of the ex vivo study, our results demonstrate the technical feasibility and procedural stability of the r‐CAIS technology, workflow, and surgical protocol. This suggests a high degree of accuracy for robotic‐assisted quad ZI surgery. Accurate implant placement prevents damage to sensitive anatomical structures and facilitates the prosthetic workflow [39]. The authors assume that the significant alignment between the planned and placed implants noted in this study may allow for efficient chairside delivery of a digitally pre‐fabricated interim prosthesis with minimal adjustments, thereby decreasing the amount of relining material required as previously published [39].

This study reported mean global platform and apex linear deviations were 1.16 mm and 1.51 mm, with an angular deviation of 1.04°. These findings have further improved in a significant manner the accuracy reported by a recent study using the same d‐CAIS system empowering the currently investigated robot, with the major difference that the authors investigated the r‐CAIS accuracy for quad ZI surgery and not for conventional implants [17]. Such positive outcomes may be related to the firm robotic guidance, that after engaging the 3D coordinates of each implant, locks the robotic arm along the implant trajectory, while the associated d‐CAIS system consistently follows the robotic execution with a dynamic live‐tracking, and quickly adjusts the implant drilling in case any deviation may occur. The most critical axis was Y (mesio‐distal) with mean deviations of −0.46 mm (SD 0.68) at platform and −0.53 mm (SD 0.84) at apex level. Maximum deviation was recorded at apex on the *Y* axis of −2.69 mm.

The secondary objective was to evaluate the potential influence of implant position (anterior vs. posterior) and quadrant (right vs. left) on global platform, global apical, and angular accuracy. The null hypothesis, which posited no differences in deviation across these variables, was partially rejected. Implant position exerted a statistically significant effect on global platform deviation (*p* = 0.0218), with posterior implants demonstrating superior accuracy. A significant association was also observed between implant position and angular deviation (*p* = 0.0465), favoring anterior implants. Conversely, none of the investigated variables had a statistically significant effect on global apical deviation.

The optically driven, seven‐joint haptic‐assisted robotic guidance system ensured consistent surgical accuracy and offered unrestricted ergonomic adaptability, irrespective of whether the procedure was performed on the right or left zygomatic bone. In Haptic and Resistance Assistance Mode, the robotic arm demonstrated a unique capability to rotate beyond the constraints of human motion, thereby facilitating precise alignment of the straight handpiece with complex three‐dimensional drilling trajectories. This optimization of the surgical approach is made possible by the system's unrestricted ergonomic range, which enhances both accuracy and operator efficiency.

The authors assumed posterior implants performed better on global platform deviation because the bone entry point is more perpendicular compared to the anteriors that, according to the quad zygomatic approach, require a more oblique entry point. Anterior implants performed better on angular deviation due to a more favorable implant trajectory, crossing stable anatomical planes. On the other hand, posterior implants show a more critical trajectory that crosses the palate first and then the zygomatic ramp, that acts as a slipway, sliding the implant laterally.

This finding emphasizes the need for surgical tools that improve the robotic arm's steady guidance, minimizing the sliding of drill tips along angled bone surfaces such as the palatal vault or lateral wall of the maxilla before entering the sinus cavity. Therefore, employing a precision drill with a sharp tip and effective side‐cutting capability as the initial drill can minimize such mechanical drawbacks and optimize the results of robotic guidance.

In the present ex vivo model, the authors deliberately selected a straight (0°) zygomatic implant design. Although angled (45°) zygomatic implants have been more frequently used in clinical practice, the primary aim of this study was the validation of surgical trajectory execution and placement accuracy within a semi‐autonomous robotic system. From a technical standpoint, the use of a straight implant design was intended to optimize mechanical stability during insertion and to reduce potential variability at the implant–mount–handpiece interface when placing long zygomatic implants. Based on the authors' clinical experience, one shortcoming of the 45° offset platform is the reduced stability of the mounter and the connection to the handpiece. The “wobbling” of these components during implant insertion might compromise the accuracy of the long zygomatic implant. Zero‐degree zygomatic implants have a more secure internal connection for the implant mounter, and this is beneficial for achieving an accurate implant insertion along the drilling coordinates. However, this observation represents expert opinion and has not been formally validated. The present experimental setup could be replicated using angled zygomatic implants to allow direct comparison in future studies.

The accuracy analysis was based on superimposing specific implant library geometries onto the silhouettes of placed implants from postoperative CBCT scans. The accuracy assessment method, even though it is the same validated procedure used in other accuracy studies, was carried out by a single well‐trained operator. Future studies addressing the intra‐ and inter‐operator variability in the digital alignment procedure may be necessary to confirm its robustness [39].

A robotic system based upon an optical dynamic navigation system offers several advantages and disadvantages compared to purely mechanical robotic systems. One key advantage is its ability to utilize dynamic navigation's calibration and patient registration tools, allowing for accurate registration using anatomical landmarks, edentulous fiducials, and thermoplastic clips with fiducials, among others. Additionally, by directly tracking the patient and the tool trackers, the system can effectively correct and bypass any mechanical error accumulation that typically occurs in the joint, encoders, and linkage of a mechanical arm. This capability reduces the errors that would arise if a mechanical sensing arm were used to track patient motion. However, a significant disadvantage is that if the navigation assembly's line‐of‐sight to the patient tracker or tool tracker is obstructed, the robotic assistance is disrupted until visibility is restored.

Another advantage associated with the optical dynamic navigation system of this robotic system is the.

AI‐assisted registration of fiducial markers. However, fiducial‐based registration accuracy may be influenced by several sources of error, including CT‐based detection, mechanical registration, and optical tracking. CT detection errors may arise from metal‐related artifacts or image distortions, particularly in the presence of multiple metallic objects or when metal artifact reduction algorithms are applied. However, metallic fiducials provide high radiographic contrast and a well‐defined spatial geometry, which can facilitate reliable and potentially sub‐voxel localization compared with lower‐density or anatomical reference points. Mechanical registration errors may derive from manufacturing tolerances of the fiducial screws or probe tip, as well as from wear or contamination of the contact surfaces. Optical tracking errors are inherent to all navigation‐based systems and are largely independent of the fiducial type. Collectively, these errors tend to propagate proportionally with distance from the fiducial cluster center. Nevertheless, the cumulative effect of these deviations is expected to be smaller than that associated with anatomy‐based registration methods, in which the primary source of error arises from human interpretation and subjective judgment when matching anatomical landmarks between the CT data and the intraoperative anatomy. In contrast, fiducial‐based, AI‐driven registration is generally associated with reduced operator‐dependent variability [37], thereby supporting its suitability for high‐precision robotic‐assisted zygomatic implant surgery.

With regard to the use of only three self‐drilling titanium screws as fiducials for the registration protocol, consistent with previously published protocols employing the same technology [39], earlier studies—such as Fan et al. [38]—reported the use of at least five fiducials for navigated zygomatic implant placement. Although a three‐point configuration is mathematically sufficient to define a rigid transformation, it may be susceptible to geometric instability and limited spatial distribution. However, such evidence refers to less advanced and technically less sophisticated registration software. In the present robotic system, the three‐point registration approach is supported by a novel AI‐driven algorithm that enables probe tracing along the anatomical surface and provides visual confirmation of registration accuracy at locations distant from the fiducials. In addition, the software allows registration refinement through the incorporation of supplementary anatomical landmarks in cases of insufficient screw stability. This backup protocol enables any anatomical point on the extensive maxillary–zygomatic surface to be digitally defined as a fiducial and subsequently identified on the patient's bone using a dedicated probe guided by AI‐assisted registration.

Furthermore, the geometry of the fiducial screws is encoded and automatically recognized during AI‐driven segmentation and pre‐registration, with the corresponding mesh accurately superimposed in the x‐, y‐, and z‐coordinate system. During registration, simply contacting the screw head with the probe tip is sufficient to localize the fiducials and complete the process with high precision.

Overall, this novel AI‐assisted registration protocol contributes to reduced preoperative surgical invasiveness by allowing the use of only three self‐drilling titanium screws instead of the five previously reported [38], with the potential to further decrease the number of required fiducials following appropriate validation.

To the authors' knowledge, no systematic reviews have specifically examined the use of robotic surgery for ZI placement. A meta‐analysis by Traboulsi‐Garet et al. compared in vitro ZI placement performed using free‐hand, s‐ and d‐CAIS systems. The study reported a mean apex deviation of 2.07 mm with d‐CAIS, 1.29 mm with s‐CAIS, and 4.98 mm for freehand placement. Angular deviations were also low for both s‐CAIS and d‐CAIS, with means of 1.07° (0.91–1.23) for s‐CAIS and 2.65° (2.55–2.72) for d‐CAIS [40]. Furthermore, Fan et al. compared in vivo the accuracy of ZI placement assisted by d‐CAIS and s‐CAIS to those performed free‐hand approach [41]. They reported linear deviations of 1.81 at the entry point and 2.95 mm at the apex point, with angular deviations of 3.49 degrees for d‐CAIS. While s‐CAIS showed 1.19 mm at the entry point and 1.80 mm at the apex point, with angular deviations of 2.15 degrees. The free‐hand group reported 2.04 mm at the entry point and 3.23 mm at the apex point, with angular deviations of 4.92 degrees [41].

In this study, r‐CAIS demonstrated superior accuracy compared to s‐CAIS and d‐CAIS as well as free‐hand for quad ZI surgery, suggesting the potential of robotic‐assisted surgical procedures for such advanced cases. This aligns with growing evidence supporting robotic systems in dental implantology. Recent systematic reviews confirm that r‐CAIS achieves higher accuracy in conventional implant placements, notably decreasing angular deviations. For instance, Luo et al. [42] reported average global platform, apex, and angular deviation of 0.69 mm, 0.72 mm, and 1.62°, respectively. Pozzi et al. [31] found that autonomous r‐CAIS could achieve at the implant level mean accuracy of 0.60 mm, at platform and 0.63 mm, at apex and 1.24° in angular deviation, suggesting improved surgical predictability and operator performance.

However, these results are not directly comparable to ZIs due to their procedural complexity and anatomical challenges. There is limited research on r‐CAIS accuracy for ZIs. Deng et al. [43] reported a prospective study with 8 ZIs, demonstrating a global coronal deviation of 0.97 mm, apical deviation of 1.27 mm, and 1.48° angular deviation. Chen et al. [44] found similar accuracy using a task‐autonomous robot, noting angular deviations of 0.92° (SD 0.40), coronal deviation of 0.48 mm (SD 0.25), and apical deviation of 0.88 mm (SD 0.28).

The findings indicate that the semi‐autonomous robotic system achieved deviations that were comparable to, or in some cases lower than, those reported for fully autonomous systems. Importantly, implant position exerted a significant influence on accuracy: anterior implants demonstrated greater global platform deviations compared to posterior implants, whereas posterior zygomatic implants exhibited higher angular deviations relative to anterior ones. These differences are likely attributable to anatomical and trajectory‐related factors, as well as implant length. Specifically, anterior ZIs were longer than posterior ZIs and their drilling and insertion path start in a more oblique on the coronal anatomic plane. The posterior ZIs performed better at the entry point because of a more vertical drilling and insertion path; however, their position in relation to the sinus cavity and their point of entrance in the zygomatic bone might affect accuracy.

Despite the r‐CAIS's Haptic Resistance mode locking the drilling trajectory, drill tips may slide along the zygomatic ramp's outer contour before entering the sinus cavity. It is therefore recommended to use ZI drills with side cuts to actively cut the bone on the side, limiting this lateral shifting of the drill tip in extrasinus ZI surgery and further optimizing robotic guidance.

The findings of this study suggest that neither the position nor the quadrant significantly impacted the accuracy of apex position of ZIs, confirming what has been reported by Sadilina et al. that robotic‐assisted surgery can mitigate human error [45]. Furthermore, the consistent results observed between the right and left upper quadrants imply that the robotic arm's physical positioning does not negatively impact implant placement or the surgeon's positioning. These conclusions highlight the potential of robotic systems to enhance surgical accuracy and consistency. However, it is imperative that future research validate these findings through in vivo studies. Such research should address the integration of robotics into surgical workflows, the duration of procedures, the learning curve associated with adopting robotic technology, and cost‐effectiveness [46]. A comprehensive evaluation of these factors will be vital in determining the practical implications and feasibility of widespread adoption of robotic assistance in surgical practices.

## 5 Conclusions

Based on the findings of this ex vivo study, the following conclusions were drawn:

1. The optically driven haptic‐assisted robotic system demonstrated high accuracy for quad ZI surgery, with mean global platform and apical deviations of about 1 mm and 1.5 mm, respectively, and an angular deviation of 1°.
2. The r‐CAIS for ZI surgery demonstrated accuracy within the range of deviations reported for current autonomous and semi‐autonomous robotic systems.
3. The r‐CAIS technology, workflow, and surgical protocol demonstrated technical feasibility and procedural stability. All ZIs were placed without significant deviations potentially affecting adjacent anatomical structures, even in an ex vivo setting. The experienced deviations can contribute to providing a preliminary mean safety margin for the 3D planning process of 1–1.5 mm and 1°.
4. The semi‐autonomous haptic robotic guidance demonstrated high procedural stability and consistency, suggesting potential to enhance operator control and predictability in complex surgeries, particularly for patients with atrophic conditions.
5. Comprehensive knowledge of advanced digital planning and further research is necessary to confirm these positive outcomes in vivo.

## Author Contributions

A.P., J.C. conceived study aims and design; A.P., J.C., A.L., A.L., collected the data; A.N. analyzed the data; A.P., J.C., A.L., I.C., A.L., and H.‐L.W. led the writing.

## Funding

The authors have nothing to report.

## Conflicts of Interest

The authors declare no conflicts of interest.

## Data Availability

The data that support the findings of this study are available on request from the corresponding author. The data are not publicly available due to privacy or ethical restrictions.
